# Automatic de-identification of textual documents in the electronic health record: a review of recent research

**DOI:** 10.1186/1471-2288-10-70

**Published:** 2010-08-02

**Authors:** Stephane M Meystre, F Jeffrey Friedlin, Brett R South, Shuying Shen, Matthew H Samore

**Affiliations:** 1 Department of Biomedical Informatics, University of Utah, Salt Lake City, Utah, USA; 2 IDEAS Center SLCVA Healthcare System, Salt Lake City, Utah, USA; 3 Medical Informatics, Regenstrief Institute, Inc., Indianapolis, Indiana, USA

## Abstract

**Background:**

In the United States, the Health Insurance Portability and Accountability Act (HIPAA) protects the confidentiality of patient data and requires the informed consent of the patient and approval of the Internal Review Board to use data for research purposes, but these requirements can be waived if data is de-identified. For clinical data to be considered de-identified, the HIPAA "Safe Harbor" technique requires 18 data elements (called PHI: Protected Health Information) to be removed. The de-identification of narrative text documents is often realized manually, and requires significant resources. Well aware of these issues, several authors have investigated automated de-identification of narrative text documents from the electronic health record, and a review of recent research in this domain is presented here.

**Methods:**

This review focuses on recently published research (after 1995), and includes relevant publications from bibliographic queries in PubMed, conference proceedings, the ACM Digital Library, and interesting publications referenced in already included papers.

**Results:**

The literature search returned more than 200 publications. The majority focused only on structured data de-identification instead of narrative text, on image de-identification, or described manual de-identification, and were therefore excluded. Finally, 18 publications describing automated text de-identification were selected for detailed analysis of the architecture and methods used, the types of PHI detected and removed, the external resources used, and the types of clinical documents targeted. All text de-identification systems aimed to identify and remove person names, and many included other types of PHI. Most systems used only one or two specific clinical document types, and were mostly based on two different groups of methodologies: pattern matching and machine learning. Many systems combined both approaches for different types of PHI, but the majority relied only on pattern matching, rules, and dictionaries.

**Conclusions:**

In general, methods based on dictionaries performed better with PHI that is rarely mentioned in clinical text, but are more difficult to generalize. Methods based on machine learning tend to perform better, especially with PHI that is not mentioned in the dictionaries used. Finally, the issues of anonymization, sufficient performance, and "over-scrubbing" are discussed in this publication.

## Background

Confidentiality of the information confided by a patient to a healthcare provider has been a cornerstone of the trust relationship established between them for centuries, as expressed in the Hippocratic Oath in ancient Greece: "...All that may come to my knowledge in the exercise of my profession or in daily commerce with men, which ought not to be spread abroad, I will keep secret and will never reveal." [1]. Certainly with increased use and adoption of Electronic Health Records (EHR) systems, greater amounts of readily accessible patient data is available for use by clinicians, researchers, and operational purposes. As data become more accessible, protecting patient confidentiality is a requirement and expectation that should not be overlooked or understated.

**Figure 1:**
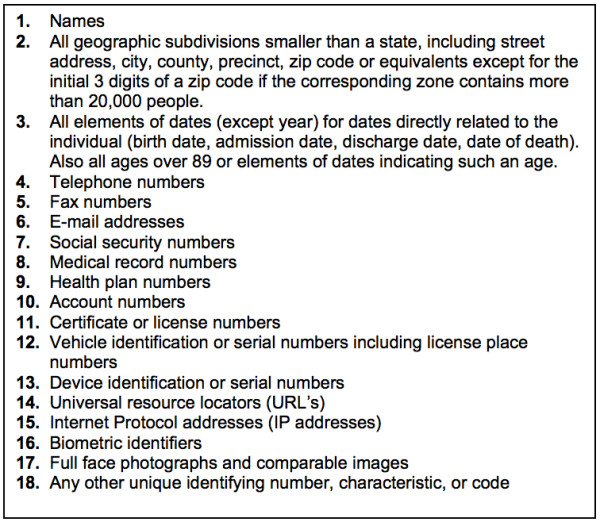
**Patient identifiers defined in the HIPAA "Safe Harbor" legislation**.

In the United States, the Health Insurance Portability and Accountability Act (HIPAA; codified as 45 CFR §160 and 164) protects the confidentiality of patient data, and the Common Rule [2] protects the confidentiality of research subjects. These laws typically require the informed consent of the patient and approval of the Internal Review Board (IRB) to use data for research purposes, but these requirements are waived if data is de-identified, or if patient consent is not possible (e.g., data mining of retrospective records). For clinical data to be considered de-identified, the HIPAA "Safe Harbor" technique requires 18 data elements (called PHI: Protected Health Information) to be removed as shown in Figure 1[3].

Anonymization and de-identification are often used interchangeably, but de-identification only means that explicit identifiers are hidden or removed, while anonymization implies that the data cannot be linked to identify the patient (i.e. de-identified is often far from anonymous). Scrubbing is also sometimes used as a synonym of de-identification. The de-identification of narrative text documents is often realized manually, and requires significant resources. Dorr et al. [4] have evaluated the time cost to manually de-identify narrative text notes (average of 87.2 ± 61 seconds per note), and concluded that it was time-consuming and difficult to exclude all PHI required by HIPAA. Already well aware of these issues, several authors have investigated automated de-identification of narrative text documents from the EHR, and many are described in details and analyzed below.

Building repositories of de-identified clinical texts has profound implications, particularly for organizations that have data warehouses containing vast amounts of legacy data available in the form of both structured and unstructured clinical data. In these systems the creation of a repository of de-identified patient data should have significant implications for the future of clinical and health services research and facilitate development of applications that have great potential to improve patient care that previously did not exist. Such repositories will provide researchers and operations with greatly increased access to patient data, thereby combining sources of data previously unavailable. For some health care systems that have large and existing EHR systems, use of combined structured and unstructured data sources for such purposes is just beginning.

The Department of Veteran's Affairs (VA) has recently funded a new informatics initiative called the Consortium for Healthcare Informatics Research (CHIR), focusing on utilizing both structured and unstructured data previously unavailable for research and operational purposes. These efforts have also focused on creating a high-performance computing environment to support data management, analytics and development environments called the Veterans' Informatics, Information and Computing Infrastructure (VINCI). Evaluating existing de-identification methods and potentially building and evaluating new methods and tools is one of the cornerstones of this initiative. Realized efforts will fulfill the ethical and legal obligations of patient privacy and confidentiality.

## Methods

Here we present a review of recent research in automatic de-identification of narrative text documents in the EHR. A comprehensive review of all research about text automatic de-identification is beyond the scope of this paper. Our review focus is only on recently published research after 1995. We selected relevant publications from bibliographic queries in PubMed (for "de-identification", "de-identification", "anonymization", and "text scrubbing"), conference proceedings, and the ACM Digital Library (for the same terms used in PubMed with "medical" or "medicine" or "biomedical" or "clinical"). We also added interesting publications referenced in papers that were already included.

## Results

The literature search resulted in a collection of more than 200 papers. A significant proportion of these publications focused only on structured data instead of narrative text. Several publications described radiological or face images de-identification, and some mentioned manual de-identification processes. Finally, 18 publications described automated text de-identification and were selected for detailed analysis of the architecture and methods used, the types of PHI detected and removed, the external resources used, and the types of clinical documents targeted. These applications are listed in Table 1 and analyzed below. They are described in subsequent sections.

**Table 1:** Automatic de-identification systems and their principal characteristics

1st author	System Name	Availability/License	**Programming language/Resources **(when known)	Knowledge resources	Document Types
Aramaki [23]	System for the i2b2 de-identification challenge	Not publicly available	CRF++^1^	Lists of names, locations, dates	Discharge summaries

Beckwith [14]	HMS Scrubber	Open source (GNU LGPL v2)	Java, JDOM, MySQL	Lists of names, locations	Surgical pathology reports

Berman [5]	Concept-Match	System freely available	Perl	UMLS Metathesaurus	Surgical pathology reports

Fielstein [7]	(VA system)	Not publicly available	Perl	Lists of names, locations, email addresses	VA compensation and pension examinations

Friedlin [8]	MeDS	Not publicly available	Java	Lists of names, locations, medical terms	HL7 messages

Gardner [24]	HIDE	Open source (Common Public License v1)	Perl, Java, Mallet ^2^	None	Surgical pathology reports

Guo [25]	System for the i2b2 de-identification challenge	Not publicly available	GATE ^3^ (ANNIE, JAPE), Java, SVM^light ^^4^	Lists of locations, hospitals.	Discharge summaries

Gupta [15]	DE-ID (DE-ID Data Corp., Richboro, PA)	Commercial system, not freely available.	Unknown	List of U.S. census names, user defined dictionaries	Surgical pathology reports

Hara [27]	System for the i2b2 de-identification challenge	Not publicly available	C++, BACT and YamCha ^5^	None	Discharge summaries

Morrison [18]	MedLEE	Not publicly available	Prolog	MedLEE lexicon, UMLS Metathesaurus	Outpatient follow-up notes

Neamatullah [9]	(MIT system)	Open source (GNU GPL v2)	Perl	Lists of common English words (non-PHI), terms indicating PHI, names and locations, known PHI (patients and staff list!)	Nursing progress notes, discharge summaries

Ruch [19]	MEDTAG framework-based	Not publicly available	Unknown	MEDTAG lexicon (based on UMLS Metathesaurus; only in French)	Various clinical documents (multilingual)

Sweeney [20]	Scrub	Not publicly available	Unknown	Lists of area codes, names	Various clinical documents

Szarvas [28]	System for the i2b2 de-identification challenge	Not publicly available	Weka ^6^	Lists of first names, locations, diseases, non-PHI (general English)	Discharge summaries

Taira [30]	(UCLA system)	Not publicly available	Unknown	List of names, and drugs	Various clinical documents

Thomas [33]	(Regenstrief Institute system)	Not publicly available	Java, XSL	List of names, UMLS Metathesaurus terms.	Surgical pathology reports

Uzuner [31]	Stat De-id	Not publicly available (open source release planned).	LIBSVM ^7^	MeSH terms, lists of names, locations, and hospitals.	Discharge summaries

Wellner [32]	System for the i2b2 de-identification challenge	Open source (BSD)	Ocaml ^8^, Carafe ^9^	Lists of US states, months, common English words.	Discharge summaries

^1 ^http://crfpp.sourceforge.net/

^2 ^http://mallet.cs.umass.edu/

^3 ^http://gate.ac.uk/

^4 ^http://svmlight.joachims.org/

^5 ^http://www.chasen.org/~taku/software/

^6 ^http://www.cs.waikato.ac.nz/ml/weka/

^7 ^http://www.csie.ntu.edu.tw/~cjlin/libsvm

^8 ^http://caml.inria.fr/ocaml/index.en.html

^9 ^http://sourceforge.net/projects/carafe/

### Identifying information and types of clinical documents processed

The automatic de-identification systems analyzed here often targeted only several types of identifying information, and not all 18 classes of PHI cited in HIPAA. Considering the format and content of each type of PHI, we classified each in seven general classes used in Table 2 and listed below:

**Table 2:** Types of PHI and other data detected by the de-identification systems

De-identification system	PHI							Clinical data
		
	Person names	Ages > 89	Geographical locations	**Hospitals/HC org**.	Dates	Contact information	IDs	
Aramaki	**P+D**	✔	✔	✔	✔	✔	✔	None

Beckwith	**P+D**	✔	✔	✔	✔	✔	✔	None

Berman	✸	✸	✸	✸	✸	✸	✸	UMLS

Fielstein	**P+D**	**-**	✔	✔	✔	✔	✔	None

Friedlin	**P+D**	✔	✔	✔	✔	✔	✔	None

Gardner	**P**	✔	**-**	**-**	✔	**-**	✔	None

Guo	**P+D**	✔	✔	✔	✔	✔	✔	None

Gupta	**P+D**	✔	✔	✔	✔	✔	✔	None

Hara	**P+D**	✔	✔	✔	✔	✔	✔	None

Morrison	✸	✸	✸	✸	✸	✸	✸	MedLEE

Neamatullah	**P+D**	✔	✔	✔	✔	✔	✔	None

Ruch	**P+D**	**-**	**-**	✔	✔	✔	✔	MEDTAG

Sweeney	**P+D**	✔	✔	✔	✔	✔	✔	None

Szarvas	**P+D**	✔	✔	✔	✔	✔	✔	None

Taira	**P**	**-**	**-**	**-**	**-**	**-**	-	None

Thomas	**P+D**	**-**	**-**	**-**	**-**	**-**	-	None

Uzuner	**P+D**	**-**	✔	✔	✔	✔	✔	None

Wellner	**P+D**	✔	✔	✔	✔	✔	✔	None

✸ Only extracted concepts (i.e. UMLS or other clinical concepts) are retained.

P+D = Patient and healthcare provider names; P = Patient name

• Person names: names of patient, family member, patient proxy, and healthcare provider.

• Ages > 89: age greater than 89.

• Geographical locations: locations such as addresses, street names, zip codes, and cities.

• Hospitals and healthcare organizations: healthcare facilities, laboratories, and nursing homes.

• Dates: all elements of dates smaller than a year.

• Contact information: phone numbers, pager numbers, fax numbers, and e-mail addresses.

• IDs: Social security number, medical record number, driver's license number, and other identifiers.

As seen in Table 2, all de-identification systems aimed to identify and remove person names (always patient names, often also healthcare provider names). Even though the majority of systems target multiple PHI types, ages over 89, geographical locations, and hospitals/healthcare organizations are most frequently ignored by de-identification systems (4 out of 18 systems). Three systems not only tried to identify PHI, but also identified clinical data and disambiguated it from PHI. This would prevent misclassifying clinical data as PHI and removing clinically relevant information during the de-identification process. One of these even removed everything but clinical data [5]. More systems (8 out of 18) used some medical knowledge to improve their detection and classification of terms as PHI, or non-PHI.

Evaluation of de-identification tasks across varied document sources is important to demonstrate generalizability of methods. However, most systems referenced in this review paper used only one or two specific document types, such as pathology reports and discharge summaries, and only three systems evaluated performance across a more heterogeneous document corpus (Table 1). The most common types of clinical text targeted by these systems-discharge summaries and surgical pathology reports-are often dictated and are composed deliberately for clear communication, while other texts like progress notes are written mainly for documentation purposes [6]. The former are "cleaner", often more structured, and therefore an easier target for automatic de-identification. It is important to note that one system used HL7 messages as a document source. This can be seen as a novel source of input data that leverages the accepted healthcare messaging standard. Even though discharge summaries are more likely to have more sensitive PHI elements, it is difficult to draw conclusions as to the accuracy and generalizability of methods with more heterogeneous document sources and less prevalent PHI elements. In order for these methods to be implemented in some practical and standardized way across operational practice, these methods should be evaluated on a larger scale. Such an evaluation should at a minimum include the diverse continuum of document types that exists in most EHR systems. Considering the large number of different clinical document types, their variability across institutions, specialties, note types, and even individuals, and the variable prevalence of PHI, this evaluation would certainly require significant resources. In a large and diverse organization such as the VA, the 100 most common clinical document types represent 80% of all clinical documents. Such a sample would probably be sufficiently representative of all clinical documents at the VA, and would include varied document types such as nursing notes, discharge summaries, physical therapy notes, echo reports, operation reports, specialized consult notes, history and physical exam notes, radiological imaging reports, etc.

### Methods used for automatic de-identification

**Figure 2:**
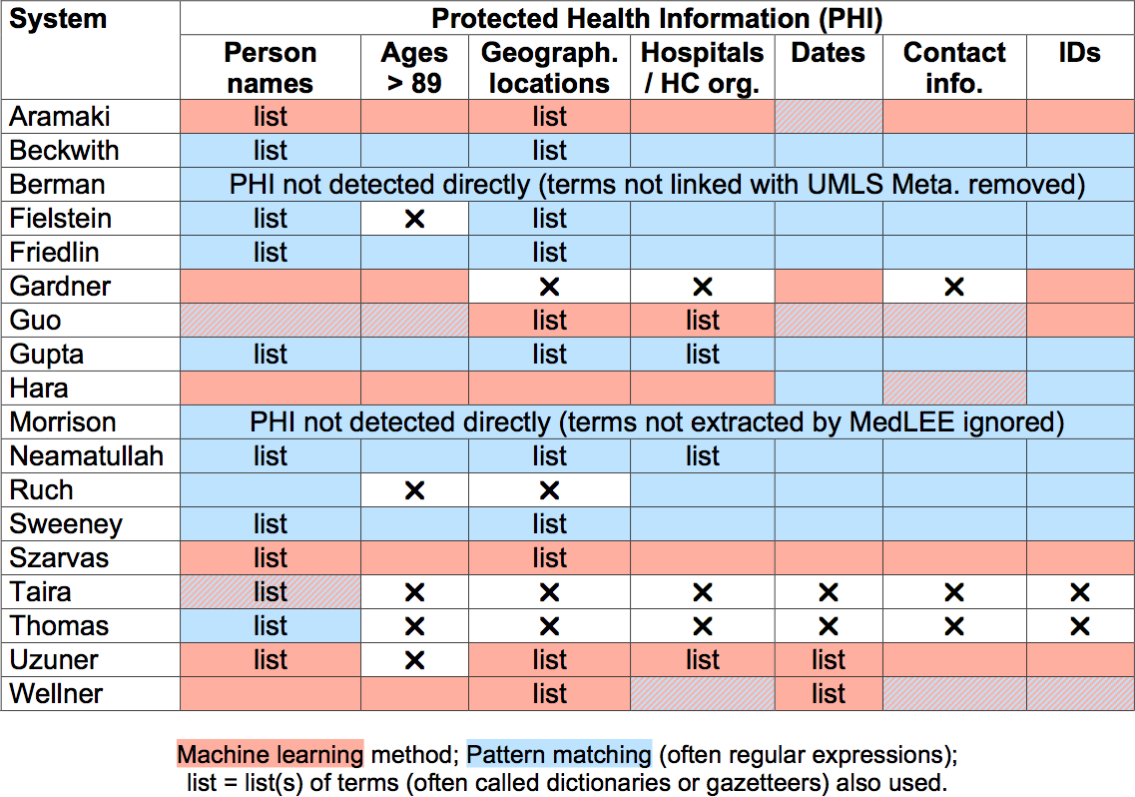
**Principal methods used for each class of PHI**.

Automated text de-identification applications are mostly based on two different groups of methodologies: pattern matching and machine learning. Many systems combine both approaches for different types of PHI (Figure 2), but the majority uses no machine learning and relies only on pattern matching, rules, and dictionaries.

These patterns, rules, and dictionaries listed in Table 3 are typically manually crafted, at the cost of months of work by experienced domain experts, and with limited generalizability. Almost all pattern matching is implemented as regular expressions, up to 50 or more for several systems. Dictionaries are built from various sources, and can be broadly classified as "PHI-like" or "not PHI-like". The first class of dictionaries list terms that are typically considered PHI, such as proper names, geographical locations, healthcare institution names, and sometimes even actual names of patients or healthcare providers of the institution in which the system was developed in [7-9]. These lists were built from publicly available resources such as the Social Security Death Index, spell-checking lexicons that include proper names (e.g., Ispell), or lists of U.S. geographical cities, counties, and states. The second class of dictionaries list terms that are in general not considered PHI, and include general English terms or biomedical terms. General English terms are provided by publicly available resources like Atkinson's words list [10] or the Ispell lexicon (after removal of proper names). Biomedical terms come mostly from the UMLS Metathesaurus [11], or from other standard terminologies like MeSH [12].

**Table 3:** Resources used by systems mostly based on pattern matching and/or rule-based methods.

De-identification system	Knowledge resources	Principal methods
Beckwith	Lists of proper names, locations	Regular expressions and dictionaries.

Berman	UMLS Metathesaurus, stop words	Dictionaries

Fielstein	Lists of cities and VA PHI (patient names, SSNs, MRNs...)	Regular expressions and dictionaries.

Friedlin	Lists of names (including Regenstrief patients), locations.	Regular expressions and dictionaries; identifiers in HL7 messages.

Gupta (De-ID system)	UMLS Metathesaurus, institution-specific identifiers	Regular expressions and dictionaries; identifiers in report headers.

Morrison (MedLEE)	MedLEE lexicon and UMLS Metathesaurus.	Rules/grammar-based, with dictionaries.

Neamatullah	Lists of common English words (non-PHI), names, locations, UMLS Metathesaurus and other medical terms, known patients and healthcare providers in the institution.	Regular expressions and dictionaries.

Ruch	MEDTAG lexicon (enriched with healthcare institution names, drug names, procedures, and devices)	Rule-based, with dictionaries.

Sweeney	Lists of names, U.S. states, countries, medical terms.	Rule-based, with dictionaries.

Thomas	List of names, UMLS Metathesaurus, Ispell terms.	Regular expressions and dictionaries.

The advantages to the rule-based and pattern matching de-identification methods is that they require little or no annotated training data, and can be easily and quickly modified to improve performance by adding rules, dictionary terms, or regular expressions. Disadvantages of pattern matching de-identification methods are the already mentioned requirement for developers to craft many complex algorithms in order to account for different categories of PHI, and the required customization to a particular dataset. As such, PHI pattern recognition performance may not be generalizable to different datasets (i.e. data from a different institution or a different type of medical report). Another disadvantage of pattern matching de-identification methods is the need for developers to be aware of all possible PHI patterns that can occur, such as unexpected date formats that use periods (e.g., 12.20.2001), or location patterns that use non-standard abbreviations (e.g., 'Cal' for California).

More recent applications tend to be mostly based on supervised machine learning methods to classify words as PHI or not PHI, and in different classes of PHI in the former case. The methods used range from Support Vector Machines, to Conditional Random Fields, Decision Trees, and Maximum Entropy (Table 3). A large corpus of annotated text is required to train these machine learning algorithms, a resource that also requires significant work by domain experts, even if text annotation is often considered to be easier than knowledge engineering. Annotated corpora can also be shared, such as during the i2b2 de-identification challenge [13]. Even the corpus annotation can be shared among many groups, such as during the i2b2 medication extraction challenge in 2009.

Almost all systems based on machine learning add some pattern matching to extract features for classification, or to detect specific types of PHI that tend to be regular, such as telephone numbers or social security numbers.

These systems use a variety of features for their machine learning algorithms (Table 4). Lexical features (word-level features) include attributes of lexical items or words in the text. They describe the word case, punctuation, special characters, numerical characters, and the morphology of the word. Each word is considered separately, but surrounding words are also often included as bi-or tri-grams. Word-level features are by far the most common types of features used for de-identification, and are also described as the most useful for the classification task. Syntactic features almost always include the part-of-speech (POS) provided by some freely available POS tagger. Semantic features refer to the semantic classification of word or phrase units. They include terms from dictionaries, and sometimes, semantic types. A few document-level features are also used, such as section headers or frequency of terms in the document. Finally, corpus-level features are rarely used and include word frequencies, mostly for disambiguation.

**Table 4:** Algorithms and features used by systems mostly based on machine learning methods.

De-identification system	Machine learning algorithm	Features		
		
		Lexical/morphological	Syntactic	Semantic
Aramaki	CRF	Word, surrounding words (5 words window), capitalization, word length, regular expressions (date, phone), sentence position and length.	POS (word + 2 surrounding words)	Dictionary terms (names, locations)

Gardner	CRF	Word lemma, capitalization, numbers, prefixes/suffixes, 2-3 character n-grams	POS (word)	None

Guo	SVM	Word, capitalization, prefixes/suffixes, word length, numbers, regular expressions (date, ID, phone, age)	POS (word)	Entities extracted by ANNIE (doctors, hospitals, locations)

Hara	SVM	Word, lemma, capitalization, regular expressions (phone, date, ID)	POS (word)	Section headings

Szarvas	Decision Tree	Word length, capitalization, numbers, regular expressions (age, date, ID, phone), token frequency	None	Dictionary terms (first names, US locations, countries, cities, diseases, non-PHI terms), section heading.

Taira	Maximum Entropy	Capitalization, punctuation, numbers, regular expressions (prefixes, physician and hospital name, syndrome/disease/procedure)	POS (word)	Semantic lexicon, dictionary terms (proper names, prefixes, drugs, devices), semantic selectional restrictions

Uzuner	SVM	Word, lexical bigrams, capitalization, punctuation, numbers, word length.	POS (word + 2 surrounding words), syntactic bigrams (link grammar)	MeSH ID, dictionary terms (names, US and world locations, hospital names), section headers.

Wellner	CRF	Word unigrams/bigrams, surrounding words (3 words window), prefixes/suffixes, capitalization, numbers, regular expressions (phone, ID, zip, date, locations/hospitals)	None	Dictionary terms (US states, months, general English terms).

CRF = Conditional Random Fields; SVM = Support Vector Machine; POS = Part-of-speech

The main advantages of machine learning de-identification methods are that they can automatically learn to recognize complex PHI patterns, and that developers of the system require only little knowledge of PHI patterns. Also, de-identification systems based on machine learning methods tend not to increase in complexity, and their processing speed does not slow over time, as can occur with pattern matching systems when progressively adapted to new document types or domains. The main disadvantage of machine learning methods is the already mentioned need, in the case of supervised learning, for large amounts of annotated training data. Another disadvantage of these methods is that it is sometimes difficult to know precisely *why *the application committed an error. For example, if a PHI location pattern is undetected by the application, adding more training data may or may not correct the error. And while machine learning de-identification methods are typically more generalizable than pattern matching methods, some additional annotated training is often required when applied to a new dataset.

In general, methods based on dictionaries performed better with PHI that is rarely mentioned in clinical text, but are more difficult to generalize. Methods based on machine learning tend to perform better, especially with PHI that is not mentioned in the dictionaries used. In the i2b2 de-identification challenge, systems based on machine learning with regular expression template features for all PHI categories performed best. They were followed by systems combining rules for some PHI categories with learning for others, and then by systems purely based on machine learning without regular expression template features or rules, and finally by purely rule-based systems [13].

### Clinical text de-identification applications mostly based on pattern matching and/or rule-based methods

HMS Scrubber was developed by **Beckwith **[14] and is an open source, HIPAA compliant, de-identification tool tailored for pathology reports. It was developed as a freely available open source scrubber. All of the tools used to create the scrubber were open source, and the source code is freely available at the Shared Pathology Informatics Network (SPIN). **Methods: **The scrubber performs a three-step process for removing PHI. First the reports are preprocessed and converted into an XML format (SPIN format). This format includes a header portion and a textual portion of the report. The header portion contains demographic information about the patient such as name, medical record number, date of birth and social security number; and information about the pathology report, such as the accession number and the pathology department. In the first of three scrubbing steps, the identifying information in these segments is extracted so it can be searched for within the body of the report via exact string matches. The second scrubbing step uses pattern matching with regular expressions. A total of 50 regular expressions are used to detect known patterns of PHI such as dates, telephone numbers, or social security numbers. Additionally, common suffixes and prefixes that likely represent names, such as "Dr." or "Mr." are also used to detect names during this step. In the third scrubbing step, a database of census derived person names and location names are used and exact string matching algorithms are used to replace any name from this list that are in the report. This database was also augmented with names from the researcher's local institution. The scrubber replaces PHI with a series of X's as well as the general category of the PHI that was removed. **Evaluation**: The scrubber was developed and tested using pathology reports obtained from three institutions within SPIN. The software was trained on training sets until it exhibited good performance (the exact number of reports used as the training set is not reported). 1800 new pathology reports (600 for each of the three hospitals) were used as a test set. They reported a recall of 98% (average for all types of PHI). The scrubber's precision was 43% (4671 over-scrubs; all types of PHI). Over-scrubs were primarily related to common words contained in proper names and places database. The reported processing speed was 47 reports per minute.

The concept-match scrubber was developed by **Berman **[5] and provides an alternative method from most traditional de-identification systems by extracting and removing every word from the text except words from an approved list of known non-PHI words. It is designed for pathology reports. **Methods: **This application is written in the Perl programming language and the software (including source code) and the nomenclatures used are available open source. The scrubber algorithm consists primarily of six steps. The report is parsed into sentences and words, and stop words (high frequency words i.e. the, a, for, in) are identified for preservation. All remaining words (non-stop words) are mapped to a standard nomenclature (the UMLS Metathesaurus [11]) with larger terms subsuming smaller terms. Matching report terms are replaced with their corresponding codes and terms from the nomenclature, and in a final step, all non-matching terms are replaced by a blocking tag. **Evaluation**: A surgical pathology text corpus was obtained from JHARCOLL, a public domain collection of more than half a million different phrases extracted from actual pathology reports (surgical pathology, cytopathology, and autopsy). All phrases in this collection were scrubbed. The actual precision and recall for the system was not reported, but it is assumed that the concept match method achieves high recall, because the only PHI likely to be retained in the text are identifiers that are also either stop words (e.g., Mr. And) or medical terms (e.g., Mr. Colon). The system is reported to suffer from low precision because of the large number of over-marked non-identifiers. Also, due to the scrubbing method of removing all non-mapped and nonstop words, the output of the scrubber would likely be hard to interpret and read. The system was fast: all 567,921 pathology phrases in JHARCOLL were scrubbed in 2,968 seconds (nearly 200 phrases per second).

**Fielstein **et al. describe a VA-based de-identification system that uses algorithms to identify PHI in Compensation and Pension examination reports [7]. The system extends the rule-based systems created by Sweeney and to a lesser extent, Berman. **Methods: **The Fielstein system's de-identification algorithms are written in the Perl programming language and consist in pattern matching with regular expressions. The regular expressions were created using public domain sources of e-mail addresses, person names from census, location names, and other resources. In addition, VA-specific regular expressions were created to match VA case numbers and account identifiers. The system uses known PHI related to a report and separately maintained within the VistA system. These identifiers include patient names, social security numbers, medical record numbers, compensation claim numbers, and VA sites of exam. The system looks for these specific known instances of PHI when performing scrubbing. **Evaluation**: The Fielstein system was evaluated using 69 VA joint exam reports. Four expert judges reviewed the de-identified reports and both over-scrubbing and under-scrubbing errors were noted. True positives were defined as text segments appropriately de-identified and true negatives were defined as text that was not PHI and not inappropriately scrubbed by the system. The overall sensitivity of the system was 81% and the overall specificity was 99% (averages for all types of PHI except ages over 89). After regular expression modification and the addition of a city name list, the sensitivity increased to 92%. The number and type of PHI instances in these 69 reports were not reported, and the number of over-scrubbing errors in relation to the number of true positives was also not provided.

**Friedlin **developed the MEdical De-identification System (MeDS) at the Regenstrief Institute [8]. The system is similar to the system described by Beckwith and Gupta. **Methods: **It uses regular expressions, patient specific data from report headers, and dictionaries of names of persons and locations to remove PHI. MeDS uses multiple processes to de-identify medical documents. First, it uses approximately 50 regular expressions to identify likely PHI patterns, such as numeric patterns like medical record numbers and zip codes, as well was numeric/text patterns such as addresses. It also uses regular expressions to identify tags that likely indicate patient or provider names such as "Dr." and "Mr.". MeDS also used dictionaries containing person names (obtained from census data and data from the Regenstrief EMR) and place names (obtained from census data). MeDS also extracts patient and provider names from the report header section and searches for these identifiers in the body of the report. Lastly, MeDS contains string similarity algorithms that it uses to identify likely misspelled patient names in a report. **Evaluation**: MeDS was evaluated in two separate evaluations. The first evaluation used a total of 2,400 reports, obtained from the Indiana Network for Patient Care (INPC) database, which included 1,400 laboratory reports, 800 narrative reports of multiple types and 200 mixed source reports. MeDS scrubbed 11,273 (99.06%) of the 11,380 HIPAA-specified identifiers, and 38,095 (98.26%) of the 38,768 non-HIPAA-specified identifiers (such as provider names/addresses). MeDS committed 4,012 (8% of the total) over-scrubbing errors. Manual review of a sample of the de-identified reports revealed that approximately 95% retained their readability and interpretability. The second evaluation used 7,193 surgical pathology reports obtained from the INPC database. MeDS scrubbed 79,993 (99.47%) of the 80,418 HIPAA specified identifiers in these reports, and 12,689 (96.93%) of the 13,091 non-HIPAA identifiers. The missed PHI consisted of identifier fragments and none represented a high risk for re-identification of the patient.

De-ID is a commercial application originally developed at the University of Pittsburgh and evaluated by **Gupta **[15]. De-Id Data Corp. acquired the global rights to De-ID Software in 2004, and it was released for commercial and academic license in March 2005. The National Cancer Institute (NCI) has licensed De-ID for de-identification of some of the applications in the Tissue Banks and Pathology Workspace of the NCI-sponsored Cancer Biomedical Informatics Grid (caBIG^® ^[16]). **Methods: **De-ID uses a set of rules, pattern matching algorithms and dictionaries to identify PHI in medical documents. De-ID analyzes report headers for patient demographic information, such as patient names, which it then uses in its string matching search of the body of the text. It also uses pattern-matching algorithms (presumably regular expressions although this is not stated) to detect PHI such as telephone numbers and zip codes. It uses a list of person names from the U.S. census and looks for a straight match to any member in this list within the text of a report. De-ID allows for the creation of user-defined dictionaries containing local identifiers specific to a given institution. It also uses the UMLS Metathesaurus to aid in identifying valid medical terms that should be retained within the document. De-ID replaces PHI with specific tags that indicate the category of the PHI removed, and the same PHI found repeatedly in a report are replaced consistently with the same tags. This helps to improve the readability of the de-identified report. Also, dates are replaced by date tags that allow for the retention of the time interval between two dates. **Evaluation**: There were a total of three evaluations of the De-ID system. All evaluations used surgical pathology reports obtained from the University of Pittsburgh Medical Center network. The first two evaluations used approximately 1000 reports and were used to develop and improve the system. The third evaluation consisted of 300 pathology reports and was used to evaluate the final version of the software. Only the number of false negative and false positive instances was reported, and since no report was given of the total number of PHI scrubbed in this final evaluation, precision and recall of the system could not be calculated.

**Morrison **used a general purpose natural language processor-MedLEE [17]-to de-identify outpatient clinic notes [18]. **Methods: **Similarly to the system described by Berman, MedLEE was used to identify and extract only the valid medical concepts in a report, and discarded all terms that were non-medical. MedLEE has been used to successfully extract concepts from numerous medical documents including radiology reports, discharge summaries, and visit notes. For this study, MedLEE was used without modifications. Manual review of MedLEE's output by a physician was performed to identify PHI retained in the output. **Evaluation**: The dataset consisted of 100 outpatient clinic notes created by internal medicine practitioners. Of the 809 instances of the PHI in the notes, 26 (3.2%) were detected in the output. Most of the PHI in the output was transformed into normalized medical terms. Examples of retained PHI in the output included patient names that are also findings (such as colors 'Green' and 'Brown') and modifiers such as 'Rose' - interpreted by MedLEE as meaning 'increased'. Additionally, MedLEE interpreted the ages as laboratory values, and the PHI of 'St' (an abbreviation for Street or Saint) as an ST segment measurement on an EKG.

**Neamatullah **describes a Perl-based de-identification software package [9] based on lexical look-up tables, regular expressions, and simple rules to identify PHI in medical text documents. **Methods: **The system uses four types of look-up dictionaries: 1) Names of patients and hospital staff obtained from the MIMIC II database, a large annotated database of cardiovascular signal data and clinical data from intensive care units (ICUs) in the United States; 2) A table of PHI names and locations which are also medical or common words (i.e., words that are in the UMLS Metathesaurus or a spell-checking dictionary; 3) A table of keywords and phrases that likely act as indicators for PHI ('Mr.', 'Dr.', 'hospital', 'Street' etc.); 4) A table of common words and phrases likely to be non-PHI, obtained from the UMLS Metathesaurus and the spell-checking dictionary. The scrubber uses three separate processes to replace PHI in medical reports. First, numerical PHI patterns (e.g., phone numbers) are identified with regular expressions. Second, non-numerical PHI (e.g., patient names) are identified with a combination of table look-ups, a context detection algorithm, and regular expressions. Lastly, all PHI identified by the above two processes are replaced with a tag to indicate the corresponding category of PHI. **Evaluation**: The Neamatullah system was evaluated on 2,434 nursing notes from 163 patients randomly selected from the MIMIC II database. A subset of these notes (99) were 'enriched' with manual insertion of additional instances of PHI. The nursing notes contained 1,779 instances of PHI as identified by the reference standard of 3 clinician reviewers and a 4th to adjudicate disagreements. The system missed 90 instances of PHI, with an estimated recall of 94% (average of all PHI types). The majority of missed PHI was first names, followed by last names. None of the missed names were patient names however. It was also noted that the software did not use the patient and provider name table obtained form the MIMIC II database for this evaluation. The system did commit numerous false positive errors resulting in a precision of only 75% (average of all PHI types). However, it was noted that the readability and information content of the de-identified notes was not compromised despite the low precision.

**Ruch **et al. describe a de-identification system based on the MEDTAG framework and consisting of a semantic lexicon specialized in medicine, and an original rule-based word-sense and morpho-syntactic tagger [19]. **Methods: **The method attempts to rely on markers to identify PHI, and to use the taggers when markers are ambiguous for PHI. The scrubber first tags the words in the text with part-of-speech tags. Then, a word-sense tagger identifies the class of a specific word in the phrase. Then based on these two tags, a determination of whether the word is PHI or not is made. For example, for the phrase "Doctors Smith and Jones saw the patient," the part-of-speech tagger identifies 'Doctors', 'Smith' and 'Jones' as nouns, and the word-sense tagger identifies the word 'Doctors' as an identity marker belonging to the *person *class. A recursive transition network uses the tags and actually makes the determination that a token is PHI. Additional terms were added to the MEDTAG lexicon for this study, including names of medical institutions, drug names, and medical procedure and device names, such as 'CT scan' and 'Doppler'. The researchers also added more than 40 disambiguation rules that were applied on a short string of tokens (up to five words). These rules helped resolve ambiguities that remained after the two sets of tags were created. **Evaluation**: The MEDTAG-based scrubber was evaluated on approximately 800 medical documents consisting of mostly post-operative reports, but also laboratory and test results, and discharge summaries (exact numbers of reports were not given). Most documents were in French, with only two discharge summaries written in English. The de-identified reports were manually reviewed to create the reference standard. The review found that the system removed 452 (96.8%) of the 467 instances of PHI in the corpus (PHI types didn't include ages over 89 and geographical locations). Of the 15 identifiers missed, 3 were partially removed, 4 completely missed, and 8 were removed when non-PHI tokens were removed. The types of identifiers missed were not explicitly stated. Error analysis revealed that some errors were caused by misspellings, while other errors could be corrected by adding more words to the lexicon. The investigators report that the system committed no over-scrubbing errors.

**Sweeney **describes Scrub, a de-identification system that uses multiple PHI detection algorithms competing in parallel to categorize and label PHI in text reports [20]. **Methods: **The Scrub system uses rule-based and dictionary-based algorithms to detect PHI. It employs a system of parallel PHI detectors, with each detector tasked to identify a specific category of PHI. For example, there are distinct detectors to identify first names, last names, full names, addresses, cities, states, and countries. There is a detector precedence based on the number of entities the detector is assigned to detect. For example, the 'Location' detector is tasked to detect a city, state and country pattern. Therefore, the 'Location' detection algorithm has a higher precedence than city, state, or country detectors. Each detector reports a certainty score for the assigned PHI category. The detector with the highest precedence and the highest certainty score above a given threshold prevails. The Scrub system attempts to consistently replace PHI with pseudo-PHI. For example, if the detected PHI is a date, a replacement date is inserted, based on a certain offset from the original date, using various algorithms. If the detected PHI is a first name, the system uses a hash-table look-up to replace the original name with a name from the table. The Scrub system also employs strategies to prevent reverse scrubbing, such as grouping all dates within a certain time period to the same date (i.e. the first of the month). **Evaluation**: The Scrub system was evaluated on a pediatric medical record system database consisting of 275 patient records that included 3,198 letters to referring physicians. The letters were both highly structured formal letters, as well as less structured delimited notes. The Scrub system was first evaluated using a certainty threshold of 0.7. This resulted in the detection of 99% of the PHI in the reports (average of all PHI types). When the threshold was reduced to 0.5, detection of 100% of the PHI occurred. There was no mention of the number of over-scrubs (terms falsely identified as PHI) and no details as to how the reference standard was created.

**Thomas **et al. describe a method for removing names in pathology reports by using a lexicon of proper names and a dictionary of common and clinical terms obtained from the UMLS Metathesaurus [21]. This system removes only proper names from medical reports. It does not attempt to remove other categories of PHI from medical documents. **Methods: **The system identifies proper names in reports by using an augmented search and replace algorithm. It uses two dictionaries to identify names: a dictionary of common and clinical usage words (CCUW) built with UMLS metathesaurus terms, and all non-proper words from an open source spell check dictionary called Ispell [22], and a second dictionary of proper names generated from three sources (all proper names from the Ispell dictionary, all patient and physician names from the Regenstrief Medical Record System, all names from the Social Security Death Index (nearly 65 million records)). The proper names dictionary was modified by removing duplicates and also removing all names also appearing in the CCUW dictionary. The algorithm also uses regular expressions to detect likely prefixes (e.g., 'Mr.' or 'Dr.') and suffixes (e.g., 'MD' or 'Jr.') to proper names. The system tokenizes the reports and the de-identification process proceeds as follows: if a token appears in the proper name list, it is removed. Otherwise, if the word is in the CCUW list, the surrounding words are checked for likely indicators of proper names and if found, the token is removed. If the word is in neither dictionary, then the surrounding words are examined for the presence of a proper name and, if found, the token is removed. **Evaluation**: The Thomas system was evaluated using 1,001 pathology reports randomly selected from the Regenstrief Medical Record System. These reports were manually annotated by two reviewers to identify all names and create the reference standard. The reference standard identified 7,710 proper names in the reports with 231 being in the narrative section of the report. The system identified 228 (98.7%) of the names found in the narrative section and 7,151 (92.7%) of all names. The names that were missed in the narrative section of the reports were all provider names and included one first name and two last names. No data was provided about the overall precision, recall and F-measure of the system, nor on the number of over-scrubbing errors that occurred.

### Clinical text de-identification applications mostly based on machine learning methods

**Aramaki **et al. describe a de-identification system based on a unique approach combining non-local features, such as sentence features and label consistency, with local features (mainly surrounding words) to identify PHI [23]. The system participated in the 2006 Informatics for Integrating Biology to Bedside (i2b2) de-identification challenge [13] and was one of the top performing systems. **Methods: **The Aramaki team first manually tagged all words of a training set with either a PHI tag or a non-PHI tag. The Aramaki system used a machine learning technique-conditional random fields (CRF)-to learn the relation between features and labels in this tagged training set. Two sets of learning processes were run. The first used the CRF to identify local features (surrounding words), non-local features (such as sentence length and location within a document) and extra-source features, mainly from external dictionaries. The second learning process used all of the features from the first learning process, plus four additional features dealing mostly with the prevalence of a label at the record and corpus level, to achieve label consistency. Label consistency is based upon the hypothesis that if the same PHI word or phrase appears multiple times within a document, it likely has the same label. Using non-local features (sentence length and location within a document) is based upon the observation that sentences with PHI tend to occur at the beginning or at the end of a document, and tend to be shorter in length than non-PHI containing sentences. **Evaluation**: For the i2b2 de-identification challenge, a corpus of 889 discharge summaries was de-identified and "re-identified" with realistic surrogates for this challenge. Seven teams participated and developed de-identification systems ranging from purely rule-based systems, to machine learning based systems and hybrid systems using both methodologies. Several systems that participated in this challenge are described below. The Aramaki team submitted only one run in the de-identification challenge. Compared to the other six teams, the Aramaki system performance was above average. For overall PHI detection, it placed third and achieved a precision, recall and F-measure all greater than 94%. For individual PHI categories, it achieved F-measures of ≥ 90%, except for ages (80%), and locations (70%).

The Health Information DE-identification (HIDE) system was developed by **Gardner **et al. [24]. **Methods: **Extraction of PHI is treated as a Named Entity Recognition (NER) problem and uses a machine learning method called Conditional Random Fields (CRF) for extracting identifying and sensitive attributes. CRF feature sets in the HIDE system included the previous word, next word, and things such as capitalization, the presence of special characters, or if the token was a number. One unique aspect of this work is the iterative classifying and retagging of the training corpus during the development of the system. This created a labeled training set in less time and effort than would be required if the corpus was tagged from scratch. **Evaluation**: The system was evaluated using 100 pathology reports from the Winship Cancer Institute at Emory. The PHI in these reports were first annotated manually, and then fed into the HIDE system. They evaluated the system's accuracy in extracting individual attributes (such as medical record numbers) as well as its overall accuracy. The overall accuracy reported for all attributes was 98.2% (average of patient names, ages over 89, dates, and IDs). Precision and recall were best with dates (100% for both) and its worst precision was with first names (97%) and the worst recall with ages (96.3%). No data was presented on the speed with which the system processed the reports.

**Guo **et al. also consider the task of identifying PHI in medical reports as a NER task, which in turn they view as a classification problem [25]. **Methods: **Their system used Support Vector Machines (SVM) and participated in the i2b2 de-identification challenge as one of the top performing systems. The Guo team used the open source GATE system [26], a natural language processing framework and model. They used the information extraction system called ANNIE, distributed freely with GATE, to preprocess and annotate a training set. ANNIE also has the capability to assign entity types to words or phrases (e.g., person name, date) but the authors noted that this process had to be modified since ANNIE's definition of an entity is not always identical to what is commonly defined as PHI. The SVM classifier used in this project was SVM^light^, an open source system that can be used as a GATE module. Multiple features were added empirically to the SVM classifier by the developers to achieve higher accuracy of PHI detection. These included date features, such as recognizing dates with a number/number or number-number pattern, and recognizing variations in telephone number patterns. Features were also added to recognize doctor names (mainly by adding prefix/suffix markers as features e.g., "Dr."), hospital names, ages, and locations. It is one of the few systems described here that does not use regular expressions. **Evaluation**: In the i2b2 challenge, the Guo team submitted two runs. In the first run, the SVM was trained only on local features and used no regular expressions. In the second run, they enriched their feature set and added rules as well as regular expressions, for mapping named entities to PHI. The system with non-enriched features and no mapping rules actually outperformed the enhanced system. Compared to the other six teams, the Guo system performance was below average. For overall PHI detection, the best performing run placed sixth and achieved a precision, recall and F-measure all greater than 86%. For individual PHI categories, it achieved F-measures of ≥ 90%, except for hospitals (83%), phone numbers (78%) and IDs (82%).

**Hara **et al. describe a de-identification system that uses SVMs and a text classifier to identify PHI in medical reports [27]. The system participated in the i2b2 de-identification challenge. **Methods: **The Hara system uses four processes to identify PHI in medical reports: 1) pattern matching to identify headings in reports; 2) regular expressions to identify patterns such as dates or telephone numbers 3) a sentence classifier that classifies sentences as containing PHI or not, 4) a text chunker based on SVMs that identifies PHI in categories including: location, hospital, doctor, patient, and age. The identification of headings for de-identification is somewhat unique in de-identification systems and even if developers initially thought it might be an important element to detect PHI, results showed that this feature only contributed very little to the identification of PHI. **Evaluation**: For the i2b2 challenge, the Hara team submitted three runs, the first two using different sentence classifiers, and the third bypassing sentence classification altogether. Interestingly, the run that bypassed sentence classification performed the best. Compared to the other six teams, the Hara system performance was average. For overall PHI detection, the best performing Hara system (without a sentence classifier) achieved a precision, recall and F-measure all greater than 92%. For individual PHI categories, the best performing Hara run achieved F-measures of ≥ 90%, except for locations (68%), patient IDs (88%), and phone numbers (86%).

**Szarvas **et al. describe a de-identification system that uses a machine learning NER approach to identity PHI in discharge records [28]. This system participated in the i2b2 de-identification challenge and was one of the best performing systems. **Methods: **This system used a modified version of an existing NER model previously used to detect entities in newswire articles [29]. They applied an iterative learning method based on decision trees that utilized the information contained in the structured (header) portion of a report to improve the recognition of PHI in the body of the report. The system includes a word-level classification model with a varied feature set. The feature set included orthographical features (e.g., capitalization, word length), frequency information, phrasal information (class of preceding words and common suffixes of the target token), dictionaries (such as first names or location names), and contextual information (such as sentence position or closest section heading). The system uses context defined by lexical triggers that are sorted based on the strength of their association with each PHI category as well as on their frequency of occurrence in the entire training set. The system also uses regular expressions to identify well-known patterns of PHI. Consistency of PHI labels in a record is achieved by post-processing the PHI labels to identify labels with the longest indentified matching phrase. **Evaluation**: In the i2b2 challenge, the Szarvas team submitted three runs. The best performing system run achieved a precision, recall and F-measure all greater than 96% for overall PHI detection. For individual PHI categories, the best performing run achieved F-measures of ≥ 90%, except for locations (68%).

**Taira **et al. describe a de-identification system that uses statistical models to remove patient names in medical reports from a pediatric urology practice [30]. **Methods: **The algorithm uses both lexicons and semantic constraint information to assign probabilities of a given word being a name. The system is designed to only remove patient names from reports and is not applicable to other categories of PHI. The Taira system uses a statistical based algorithm to estimate the probability of a patient name reference within the context of a predefined set of logical relations. The system processes each sentence in a report and classifies it according to the type of logical relation it contains. If the logical relation is determined to be of a PATIENT type, the potential name is extracted. The algorithm uses a lexicon of over 64,000 first and last names, as well as a set of semantic selectional restrictions that place contextual requirements upon candidate words in the report text. This is based on the hypothesis that strong associations exist between some classes of words and some classes of concepts. For example the word 'presented' may be strongly associated with the concept of patient name as in "John Smith presented to the clinic today". These constraints were determined automatically from a manually tagged corpus of training reports, and act as a template for various semantic restrictions placed on the descriptions of patient names in the training set. **Evaluation**: The Taira system was trained using 1,350 randomly selected reports from pediatric patients generated at the UCLA Clark Urology Center. Researchers manually tagged a total of 907 patient names in this corpus. The test set consisted of 900 reports from the same clinic and consisted of letters to referring physicians, discharge summaries, clinic notes, and operative reports. The system output was compared to that of the manually tagged reference standard. The area under the ROC curve was 0.97. The threshold value with the best accuracy was 0.55 and attained a precision of 99% and a recall of 94% (for patient names). Reasons for false positive errors included semantically incorrect but valid name syntax, identification of the patient relative instead of the patient, medical terms that can also be proper names, and some proper drug names. Reasons for false negative errors included logical relations that were not modeled, and grammatically difficult expressions.

Stat De-id was developed by **Uzuner **et al. and treats de-identification as a multi-class classification task [31]. It uses SVMs to classify tokens as either one of several categories of PHI, or non-PHI. **Methods: **The Stat De-id system attempts to identify the local context of a particular token in order to determine the class of the token. The system identifies orthographic, syntactic and semantic characteristics of a token by examining a ± 2 token window around the target token. The system is unique in that it augments the syntactic context by parsing the sentence containing the token using a Link Grammar Parser, which is capable of partially parsing even malformed sentences. Examples of lexical and orthographic features include the target token itself, capitalization, punctuation, and word length. Examples of syntactic features include the part-of-speech of the token, the part-of-speech of surrounding tokens, and the output of the Link Grammar Parser. For this system, they used LIBSVM, an open source integrated software library for multi-class SVMs. **Evaluation**: The Stat De-id system was evaluated using 889 discharge summaries obtained from the various medical departments of the Partners Healthcare System in Boston, MA. This was the same corpus used in the 2006 i2b2 de-identification challenge. The system achieved an F-measure of 98% with a precision of 99% and a recall of 97% (average of all PHI types).

**Wellner **et al. describe a de-identification system based on the adaptation of two toolkits for named entity recognition (Carafe and LingPipe) [32]. **Methods: **Wellner et al. treated the de-identification task as a sequence-labeling problem in which labels are assigned to individual words indicating whether the word is the beginning, part of, or end of a particular PHI instance. The system participated in the i2b2 de-identification challenge and was the best performing system. The Wellner system was developed using two different sequence-labeling systems. The first is called Carafe, a toolkit implementing Conditional Random Fields and targeted especially for phrase identification tasks. It is structured to allow for easy introduction of new features, and the researchers performed task-specific modifications of Carafe to increase de-identification accuracy. These included adding tokenization adjustments (mainly to account for date patterns). In order to ensure the system favors recall over precision, they introduced a bias parameter that adjusts model weight based on prior probability to a token to a particular label. However, introducing this bias parameter alone did not sufficiently improve recall; therefore a number of regular expressions were used during post-processing to further improve recall. The other sequence labeling system used in development was called LingPipe, a software developer's toolkit of Java classes for performing a variety of NLP tasks. For the de-identification task, the developers used LingPipes' named entity tagging feature. Based on hidden Markov models, the named entity tagging feature uses text chunking and n-gram models to make predictions about entities based on statistics gathered from training. A chunk labeled as a named entity was considered PHI. **Evaluation**: In the i2b2 challenge, the Wellner team submitted three runs, two using the Carafe system and one run using LingPipe. For overall PHI detection, the best performing system (using Carafe) achieved a precision, recall and F-measure all greater than 96%. For individual PHI categories, the best performing run achieved F-measures of ≥ 93%, except for locations (78%), ages (80%), and phone numbers (85%).

## Discussion

In this paper, we present a review of the state of the art in automated de-identification of clinical text. Methods that performed well to de-identify text include machine learning approaches based on CRF, Decision Trees, Maximum Entropy models, or SVM, combined with dictionaries and sometimes regular expressions. Though many systems report promising performance on a specific document corpus used for their evaluation, these systems should be tested on a more heterogeneous document set to assess generalizability across clinical documents, various EHR systems, and de-identification of all identifiers specified by HIPAA. These performances were also not evaluated in terms of acceptability: how good is enough? This question is largely dependent on the particular use case and document type(s). Certainly there are specific tradeoffs when evaluating one approach over another in terms of precision and recall, but there may also be other issues related to workload, and cost of practical system implementation when evaluation is conducted using more heterogeneous data sources. The approach that provides optimal results for one use case or data source may not generalize when applied to a different data source or clinical use case. Also, none of the studies in our review looked at the effects of de-identification on subsequent automated information extraction tasks, nor did they look at how automated de-identification affects the readability and interpretability of remaining clinical data.

As already mentioned, de-identified data is often not completely anonymous (i.e. the possibility that the patient could be re-identified cannot be excluded). As the amount of patient data increases across clinical domains, awareness of the types of data available and the potential risks of re-identification will increase. Discussions of regulatory issues and policy are beyond the scope of this review, but it is important to note that the policy expectations will change as data are made more widely available and that privacy and utility of patient data need not be mutually exclusive goals. The de-identification methods discussed in this review will certainly play into these decisions.

An important question is what performance could be considered sufficient to feel confident that the risk of re-identification is negligible. Is removing 90% of the dates sufficient? Is missing 10% of the names acceptable? Since the risk of re-identification related to the different types of PHI varies widely, a different sufficient performance should be defined for each type of PHI. Names should not be missed, but rare address portions like cities could probably be missed. Sufficient performance will also depend on the context in which the de-identified data will be used. Insiders, like healthcare personnel with access to local identifiers (e.g. laboratory test numbers) and knowledgeable of the social and medical history of patients, are far more likely to recognize a patient from de-identified text than external researchers with no access to any additional data about the patients. The definition of sufficient levels of performance that depend on the type of PHI and the future uses of the de-identified text are desirable, and might be part of future regulatory issues and policies.

The ultimate goal of de-identification software is to scrub true patient identifiers while minimizing over-scrubbing (erroneously removing non-PHI data). A medical report completely scrubbed of not only all patient identifiers but also most of the important medical data as well is of little use to researchers. Over-scrubbing errors can be grouped into 2 general categories: 1) Over-scrubbing that removes pertinent medical data thereby affecting the readability and interpretability of the de-identified report; 2) Over-scrubbing that has little or no effect on readability and interpretability. Removing a diagnosis, finding, or medication are examples of the first category. Removal of common words, such as 'the', 'a', 'and', and removal of non-medical data such as the phrases 'was seen', 'was examined', 'following this' are examples of the second category.

While we agree that the main focus of the evaluation of a de-identification system should be its accuracy in removing PHI from medical documents, assessing over-scrubbing errors is also important. In addition to calculating PHI removal accuracy, thoroughly evaluating over-scrubbing errors and the degree to which they affect the research value of the final de-identified reports provide valuable data in determining the practical usefulness of a de-identification system.

Often, studies involving de-identification systems focus only on the system's ability to remove PHI and give little or no detail regarding over-scrubbing errors [7,15,20,33]. Those studies that do provide over-scrubbing error data, rarely offer details on what was over-scrubbed and how the over-scrubbing affected the research value of the de-identified reports. A standard method of measuring the effects of over-scrubbing errors would be helpful in the evaluation of de-identification systems.

De-identified reports will probably be less valuable for research compared to fully identified reports. For example, dates (more specific than year) are considered PHI and therefore must be removed or altered in de-identified reports. Temporal information, such as knowledge that one report, disease, finding, or treatment occurs before or after another is often important for medical research. Also, some diseases or findings may have seasonal patterns, which could be important to researchers. Such data is generally lost when reports are de-identified. A few de-identification systems attempt to retain some temporal data by using 'date altering'-where the same random interval is applied to every occurrence of a date in an entire patient record, thereby preserving the intervals between dates [15,8]. Other data potentially valuable to researchers, for example geographic information (both specific-such as hospital or clinic names and more general such as the names of major cities), patient occupations, and ages of elderly patients older than 89, are lost through the de-identification process and not present in de-identified reports.

It is also possible that the de-identification process has an adverse effect on the accuracy of automated identification and extraction of medical concepts from de-identified documents. Technologies such as natural language processing (NLP) and text data mining applications such as those in the Statistical Analysis System (SAS) may be less effective when processing de-identified reports compared to fully identified reports. To the best of our knowledge, no studies have evaluated this issue. The i2b2 NLP challenges used de-identified discharge summaries [13,34,35]. Neither the smoking status identification challenge, nor the obesity and comorbidities detection challenge, cited the de-identification process itself as a cause of NLP failures for any of the systems involved in the challenge. However, the discharge summaries in the i2b2 challenge were de-identified using methods quite different than the automated methods used by systems mentioned in this paper, so caution must be used in concluding that de-identification has no effect on NLP system performance. In the i2b2 challenge, the de-identification was performed semi-automatically with manual review of scrubbing, and all PHI was replaced by synthetic identifiers. It is likely then that the de-identified discharge summaries used in the i2b2 challenges and the de-identified discharge summaries produced by processing the same reports using any of the systems mentioned here would be quite different.

## Conclusions

We have reviewed recent research in automatic de-identification of narrative text documents in the EHR, and analyzed the methods and resources used, as well as discussed 18 systems included in this analysis. This review will inform our evaluation of existing de-identification methods and the development of a best-of-breed automatic clinical text de-identification system in the context of the Department of Veteran's Affairs CHIR and VINCI projects. The evaluation of the level of anonymization and risk for re-identification of automatically de-identified clinical text, as well as the impact of the de-identification process on subsequent use of clinical text will also be part of these projects. These efforts will enhance the usability of clinical data for research, and fulfil the ethical and legal obligations of patient privacy and confidentiality.

## Competing interests

The authors declare that they have no competing interests.

## Authors' contributions

SMM conceived and organized the review, analyzed publications, and helped draft the manuscript. FJF, BRS, and SS analyzed publications, and helped draft the manuscript. MHS revised the manuscript critically and in details. All authors read and approved the final manuscript.

## Pre-publication history

The pre-publication history for this paper can be accessed here:

http://www.biomedcentral.com/1471-2288/10/70/prepub
